# A Quantum Algorithm Detecting Concentrated Maps

**DOI:** 10.6028/jres.112.025

**Published:** 2007-12-01

**Authors:** Isabel Beichl, Stephen S. Bullock, Daegene Song

**Affiliations:** National Institute of Standards and Technology, Gaithersburg, MD 20899; Institute for Defense Analyses, Center for Computing Sciences, Bowie, MD 20715; Korea Institute for Advanced Study, Seoul 130-722, Korea

**Keywords:** concentrated maps, Deutsch-Jozsa algorithm, Deutsch’s algorithm, one-to-one mappings, quantum computation, quantum oracle, roots of unity

## Abstract

We consider an arbitrary mapping *f*: {0, …, *N* − 1} → {0, …, N − 1} for *N* = 2*^n^*, *n* some number of quantum bits. Using *N* calls to a classical oracle evaluating *f*(*x*) and an *N*-bit memory, it is possible to determine whether *f*(*x*) is one-to-one. For some radian angle 0 ≤ *θ* ≤ π/2, we say *f*(*x*) is *θ* − *concentrated* if and only if $e^{2\pi if(x)/N}\subset e^{i[\psi_{0}-\theta ,\psi_{0}+\theta ]}$ for some given *ψ*_0_ and any 0 ≤ *x* ≤ *N* − 1. We present a quantum algorithm that distinguishes a *θ*-concentrated *f*(*x*) from a one-to-one *f*(*x*) in *O*(1) calls to a quantum oracle function *U_f_* with high probability. For 0 < *θ* < 0.3301 rad, the quantum algorithm outperforms random (classical) evaluation of the function testing for dispersed values (on average). Maximal outperformance occurs at $\theta =\frac{1}{2}\sin^{-1}\frac{1}{\pi }\approx 0.1620$ rad.

## 1. Introduction

In recent years, much progress has been made in the study of quantum computation [1,2]. The first algorithm arguing for computational speed-up due to quantum mechanics was discovered in 1985 [3]. Deutsch considered a mapping with two inputs and two outputs. An oracle, which one might think of as a classical black-box, evaluates functions of a bit by inputting *b* ∈ {0, 1} and outputting *f*(*b*) ∈ {0, 1}. Two calls to such an oracle are required to learn whether *f* is one-to-one. The calls compute *f*(0) and *f*(1), and then the one-to-one property holds when the values are distinct. Since quantum mechanics is linear, a quantum function evaluator (quantum oracle) must act on superpositions of states.

$$U_{f}\left(\alpha |0,0\rangle +\beta |1,0\rangle \right)=\alpha |0,f(0)\rangle +\beta |1,f(1)\rangle$$ (1)

A single call to this quantum oracle allows one to determine whether *f*(0) and *f*(1) are distinct [2, pg. 36]. Several years later, Deutsch and Jozsa generalized the algorithm to allow for multiple inputs and two outputs [4]. Specifically, they describe a multi-argument function as balanced if its image holds two elements and the preimage of each is the same size. Deutsch and Jozsa’s algorithm then distinguishes between a constant and balanced function using a single quantum oracle call. Further generalizations [5] distinguish between functions which are constant or else map onto an evenly spaced subset of the unit circle {|*z*| = 1}.

We present a variant of such algorithms. Specifically, suppose that we have a function f: {0, 1, 2, …, N − 1} → {0, 1, …, *N* − 1}, where *N* = 2*^n^* for *n* some (integer) number of qubits, so that the *n*-qubit state space ${ℋ}_{1}^{⊗n}$ is *N* dimensional [2]. Let *ω* = *e*^2π/^*^N^* be the (2*^n^*)th root of unity, and choose *ψ*_0_ ∈ [0, 2π). We say such an *f*(*x*) is *θ* − *concentrated* about *ψ*_0_ if and only if

$$\omega^{f(x)}\in \exp(i[\psi_{0}-\theta ,\psi_{0}+\theta ]),\;\forall 0\leq x\leq N-1$$ (2)

We say *f*(*x*) is *θ*-concentrated if and only if there exists a *ψ*_0_ so that (2) holds. Using *N* − 1 bits and *N* evaluations of the function (classical oracle calls), we may determine with certainty whether *f*(*x*) is one-to-one. Suppose instead one has a quantum oracle *U_f_* encoding an *f*(*x*) which is known to be either constant or concentrated. We here present an algorithm which uses *O*(1) calls to *U_f_* to distinguish between these cases, with arbitrarily high probability.

To describe *U_f_*, we briefly review quantum data spaces [2,6]. The state of a string of quantum bits is encoded as a vector in a complex Hilbert space, say |*ψ*〉 ∈ ℋ. For qubit-states, the usual convention is that the one-qubit state space is ${ℋ}_{1}={\text{span}}_{C}\{|0\rangle ,|1\rangle \}$, where this basis is Hermitian orthonormal. The *n*-qubit state space is then the *N* = 2*^n^* tensor (Kronecker) product

$$\begin{array}{l} {ℋ}_{n}={\text{span}}_{C}\{|b_{1}\rangle ⊗|b_{2}\rangle ⊗\cdots ⊗|b_{n}\rangle :\\ \;b_{j}\in F_{2}=\{0,1\},1\leq j\leq n\} \end{array}$$ (3)

The abbreviation |*b*_1_*b*_2_…*b_n_*〉 for |*b*_1_〉 ⊗ |*b*_2_〉 ⊗ ⋯ ⊗ |*b_n_*〉 is typical, and the Hermitian inner product is that induced by the tensor structure. At times, we further abbreviate the bit-string *b*_1_*b*_2_…*b_n_* within the ket by the associated integer, i.e., the binary expansion. Explicit description of the oracle also makes it simpler to take 2*n* to be our number of quantum bits. We then refer to a *first register and a second register*, according to the tensor decomposition ${ℋ}_{2n}={ℋ}_{n}⊗{ℋ}_{n}$.

Given this, the conventions for the quantum oracle box are as following. The oracle *U_f_* effects a unitary transformation of ${ℋ}_{2n}$ which linearly extends

$$U_{f}|x\rangle |y\rangle =|x\rangle |y⊕f(x)\rangle$$ (4)

where *y* ⊕ *f*(*x*) denotes *y* + *f*(*x*) mod *N* and the tensor symbols have been suppressed. Our quantum algorithm then requires *O*(1) calls to *U_f_* and *O*(*n*^2^) two-qubit gates otherwise to distinguish with probability arbitrarily close to one between the cases

- *f*(*x*) is one-to-one
- *f*(*x*) is *θ*-concentrated

Hence the quantum algorithm in this sense outperforms a classical device using *O*(*N*) classical oracle calls to determine whether *f*(*x*) is one-to-one with certainty. However, consider instead a probabilistic classical computer, capable of evaluating *f*(*x*) on a given random *x*, 0 ≤ *x* ≤ *N* − 1. With a single oracle call, such a classical probabilistic computer is likely to detect *f*(*x*) is not *θ*-concentrated with probability $1-\frac{2\theta }{2\pi }$. Hence *f*(*x*) is one-to-one, by hypothesis. Making use of a single quantum oracle call, our quantum algorithm identifies any one-to-one function with certainty, and it correctly identifies a *θ*-concentrated *f*(*x*) with probability cos^2^*θ*. Taking *f*(*x*) one-to-one or *θ*-concentrated, each with probability $\frac{1}{2}$, further demonstrates that the quantum algorithm outperforms the classical probabilistic algorithm on average for 0 < *θ* < 0.3301 rad, with maximal quantum outperformance at $\theta =\frac{1}{2}\sin^{-1}\frac{1}{\pi }\approx 0.1620$*θ* rad.

## 2. A Solution with No Quantum Oracle

This section applies to any *f*: {0, 1, ⋯, *N* − 1} → {0, 1, ⋯, *N* − 1} whether *N* = 2*^n^* or not. In the sequel, choosing *N* = 2*^n^* makes possible small quantum Fourier transform circuits, i.e., efficient quantum implementations of the Fourier transform of **Z**/*N***Z**.

To determine whether *f*(*x*) is one-to-one, proceed as follows. We suppose a classical oracle capable of evaluating *f*(*x*) and a memory block of size *N* bits.


Initialize each memory bit to 0
for (j=0; j<= N−1; ++j)
{ Use oracle to compute *f*(*j*)
 if[ (bit # *f*(*j*)) = 1]
 { report **not 1**−**1**
  return }
 Assign 1 to bit *f*(*j*) }
report **1**−**1**


Moreover, note that there can not exist any oracle-based algorithm which determines whether *f*(*x*) is one-to-one while only using *N* − 1 or fewer calls to the classical oracle which evaluates *f*(*x*).

Since the quantum algorithm will only decide between the one-to-one and *θ*-concentrated cases with probability very close to one, we also consider competitive probabilistic classical algorithms. For simplicity, suppose now *f*(*x*) is either one-to-one or *θ*-concentrated about 0, i.e., *ψ*_0_ = 0 in (2). Given a random number generator, the following algorithm is immediate:


Choose a random 0 ≤ *x* ≤ *N* − 1
Evaluate *f*(*x*)
if [*ω^f^*^(^*^x^*^)^ ∉ exp(*i*[−*θ*, *θ*])]
 report *f*(*x*) **is 1**−**1**
else
 report *f*(*x*) **is likely concentrated**


The probabilistic algorithm fails if and only if *f*(*x*) is one-to-one and yet *ω^f^*^(^*^x^*^)^ ∈ exp(*i*[−*θ*, *θ*]), roughly with probability $1-\frac{\theta }{\pi }$ for *n* large.

## 3. A Quantum Variant of Deutsch-Jozsa

The following algorithm exploits a quantum oracle *U_f_* per Eq. (4). It requires two quantum registers, each *n* bits long.

To distinguish a concentrated from a one-to-one *f*(*x*):

1. Prepare the first register as |0〉^⊗^*^n^* and the second as |0〉^⊗^*^n^*. Thus the original data state is |Φ〉 = |Φ_1_〉 ⊗ |Φ_2_〉 = |0〉^⊗^*^n^*|1〉^⊗^*^n^*.
2. Let ω = *e*^2π^*^i^*^/^*^N^*, for *N* = 2*n*. As is well-known [2], there is a quantum circuit, polynomial in size in *n*, which implements the quantum Fourier transform map: $ℱ={ℋ}_{n}\to {ℋ}_{n}$ linearly extending $|y\rangle \mapsto \frac{1}{\sqrt{N}}{\sum_{y=0}^{N-1}\omega }^{ya}|z\rangle$. Apply ℱ to the second register, for ${|\Phi \rangle }_{2}=ℱ|N-1\rangle =\frac{1}{\sqrt{N}}{\sum_{z=0}^{N-1}\omega }^{-z}|z\rangle$.
3. Recall the one-qubit Hadamard gate given by $H=\frac{1}{\sqrt{2}}\sum_{j,k=0}^{1}{\left(-1\right)}^{jk}|j\rangle \langle k|$. Then apply *H*^⊗^*^n^* to the first register, with the result that
  $$|\Phi_{1}\rangle ={\left(H|0\rangle \right)}^{⊗n}={\left[\frac{1}{\sqrt{2}}(|0\rangle +|1\rangle )\right]}^{⊗n}=\frac{1}{\sqrt{N}}\sum_{x=0}^{N-1}|x\rangle$$ (5)
  Thus the first register now holds an equal superposition of all states. As preparation for the next step, we also note the full data state:
  $$|\Phi_{1}\rangle ⊗|\Phi_{2}\rangle =\frac{1}{N}\sum_{x=0}^{N-1}\sum_{y=0}^{N-1}\omega^{-y}|x\rangle |y\rangle$$ (6)
4. We next apply the quantum oracle *U_f_*. The result is
  $$\begin{array}{l} |\Phi_{1}\rangle ⊗|\Phi_{2}\rangle =U_{f}\frac{1}{N}\sum_{x=0}^{N-1}\sum_{y=0}^{N-1}\omega^{-y}|x\rangle |y\rangle \\ \;=\frac{1}{N}\sum_{x=0}^{N-1}\sum_{y=0}^{N-1}\omega^{-y}|x\rangle |y⊗f(x)\rangle \end{array}$$ (7)
  Note that a single call to *U_f_* implicitly uses every value of *f*(*x*) for a state in full superposition, such as |Φ_1_〉.
5. We reindex a sum in the last equation as follows. For fixed *x* = *x*_0_, label *z* = *y* − *f*(*x*). Then *y*|*z*〉. As this is true for all *x*_0_, we have
  $$\begin{array}{l} |\Phi_{1}\rangle ⊗|\Phi_{2}\rangle =\frac{1}{N}\sum_{x=0}^{N-1}\sum_{x=0}^{N-1}\omega^{-z+f(x)}|x\rangle |z\rangle \\ \;=\left(\frac{1}{\sqrt{N}}\sum_{x=0}^{N-1}\omega^{f(x)}|x\rangle \right)⊗\left(\frac{1}{\sqrt{N}}\sum_{z=0}^{N-1}\omega^{-z}|z\rangle \right) \end{array}$$ (8)
  The next step is to disregard the known data |Φ_2_〉 in the second register.
6. Apply a Fourier transform to the retained register for
  $$\begin{array}{l} |\Phi_{1}\rangle =\frac{1}{N}\sum_{x=0}^{N-1}\sum_{y=0}^{N-1}\omega^{xy+f(x)}|y\rangle \\ \;=\left(\frac{1}{N}\sum_{x=0}^{N-1}\omega^{f(x)}\right)|0\rangle +\frac{1}{N}\sum_{y=0}^{N-1}\sum_{x=0}^{N-1}\omega^{xy+f(x)}|y\rangle \end{array}$$ (9)
7. Measure the probability that |Φ_1_〉 is |00 … 0〉. Recall that the probability of this classical outcome is its square of the amplitude (i.e., coefficient) of |00 … 0〉 in the coherent superposition |Φ_1_〉. (9),
  $$\mathrm{Prob}(|\Phi_{1}\rangle =|00\ldots 0\rangle )={\left|\frac{1}{N}\sum_{x=0}^{N-1}\omega^{f(x)}\right|}^{2}$$ (10)
8. Should the classical bits 00 … 0 be observed, assert that *f* is concentrated. Else assert that *f* is one-to-one.

We briefly comment on the quantum computational resources consumed. Besides the 2*n*-qubits, *O*(*n*) local computations and two *n*-qubit Fourier transforms are required. The latter require *O*(*n*^2^) gates [1].

How likely are the assertions of the last step to be correct? Observing |Φ_1_〉 = |00 ⋯ 0〉 has probability of zero if *f*(*x*) is one-to-one, since $\sum_{J=0}^{n-1}\omega^{j}=0$; we prove below that this observation has probability at least cos^2^*θ* if *f*(*x*) is *θ*-concentrated. Hence, to distinguish any one-to-one *f*(*x*) from a *θ*-concentrated *f*(*x*) using *U_f_* with probability 1 − *ε*, run at least *T* independent trials of the above for *ε* > sin^2^*^T^θ*. In terms of *ε*, as log sin *θ* < 0 we demand $T>\frac{1}{2}\frac{\log\varepsilon }{\log\;\sin\theta }$.

**Proposition:** Let *f*: {0, 1, …, *N* − 1} → {0, 1, …, *N* − 1}, *N* = 2*^n^* be *θ-concentrated, and continue to denote ω* = *e*^2π^*^i^*^/^*^N^*. *Then*

$$(f(x)\text{is one}-\text{to}-\text{one})\Rightarrow \left(\sum_{x=0}^{N-1}\omega^{f(x)}=0\right)$$ (11)

*Hence, the* |0〉 *coefficient of the output* |Φ_1_〉 *is* 0 *if f*(*x*) *is one-to-one. On the other hand*,

$$(f(x)\text{is concentrated})\Rightarrow \left(\left|\sum_{x=0}^{N-1}\omega^{f(x)}\right|\geq N\cos\theta \right)$$ (12)

**Proof:** First, recall that as an *N*^th^ root of unity, ω = *e*^2π^*^i^*^/^*^N^* solves *z^N^* − 1 = 0. Then

- $z^{N}-1=(z-1)(\sum_{j=0}^{N-1}z^{j})$
- ω ≠ 1
- For *f*(*x*) one-to-one, $\sum_{j=0}^{N-1}\omega^{j}=\sum_{j=0}^{N-1}\omega^{f(j)}$.

Thus Eq. (11) follows.

Suppose on the other hand that *f*(*x*) is concentrated. Then $\omega^{f(i)-i\psi_{0}}=a_{j}+ib_{j}$ for *ψ*_0_ per Eq. (2), and moreover cos*θ* ≤ *a_j_* ≤ 1.

$$\left|\sum_{x=0}^{N-1}\omega^{f(x)}\right|=\sqrt{{\left(\sum_{j=0}^{N-1}a_{j}\right)}^{2}+{\left(\sum_{j=0}^{N-1}b_{j}\right)}^{2}}\geq \sum_{j=0}^{N-1}a_{j}\geq N\cos\theta$$ (13)

This concludes the proof of Eq. (12). □

## 4. Comparison of Quantum to Classical

We finally compare the probabilistic classical algorithm with the quantum algorithm above, allowing each a single oracle call. For simplicity we suppose *ψ*_0_ = 0 in (2); this hypothesis favors the classical algorithm. Also for simplicity, we suppose *f*(*x*) is equally likely to be either concentrated or one-to-one.

Thus *f*(*x*) is either one-to-one (event *O*) or *θ*-concentrated (event *C*) with probability $\frac{1}{2}$. Suppose the classical probabilistic algorithm makes one oracle call and then guesses *f*(*x*) is concentrated if *ω ^f^*^(^*^x^*^)^ lies within the sector exp(*i*[−*θ*, *θ*]) and one-to-one else. If *f*(*x*) is *θ*-concentrated, then the classical algorithm makes a correct guess (event *G_C_*). In the one-to-one case, the probability of a correct guess is approximately $1-\frac{\theta }{\pi }$. So

$$\begin{array}{l} \text{Prob}(G_{c})=\text{Prob}(G_{C}|O)\text{Prob}(O)+\text{Prob}(G_{C}|C)\text{Prob}(C)\\ \;\approx \left(1-\frac{\theta }{\pi }\right)(1/2)+(1)(1/2)\\ \;=1-\frac{\theta }{2\pi } \end{array}$$ (14)

If multiple oracle calls are allowed, it will help to recall *x* from previous trials and force the oracle to evaluate new values. However, as *N* = 2*^n^* is expected to be large, this is a minor consideration, and $1-{\left(\frac{\theta }{2\pi }\right)}^{l}$ is approximately the probability of making a correct guess after *l*-trials.

In contrast, consider the quantum algorithm. It guesses *f*(*x*) is concentrated if |00 ⋯ 0〉 is observed and guesses one-to-one else. Thus, in contrast to the classical algorithm, the quantum algorithm never fails if *f*(*x*) is one-to-one. If *f*(*x*) is concentrated, then the quantum guess is correct with probability of at least cos^2^*θ*. Thus

$$\begin{array}{l} \text{Prob}(G_{Q})=\text{Prob}(G_{Q}|O)\text{Prob}(O)+\text{Prob}(G_{Q}|C)\text{Prob}(C)\\ \;\geq (1)(1/2)+(\cos^{2}\theta )(1/2) \end{array}$$ (15)

Thus the appropriate comparison of the probabilistic and quantum algorithms might be quantified by the difference Prob(*G_Q_*)−Prob(*G_C_*), i.e., the quantum approach is preferable for those *θ* with $\cos^{2}\theta \geq 1-\frac{\theta }{\pi }$, i.e., $\sin^{2}\theta \geq \frac{\theta }{\pi }$. The maximum difference occurs at $\theta =\frac{1}{2}\sin^{-1}\frac{1}{\pi }\approx 0.1620$ rad, while applying Newton’s method to $\sin^{2}\theta -\frac{\theta }{\pi }$ shows that the quantum approach is preferable given 0 < *θ* < 0.3301 rad. The right boundary of the interval is approximate.

## References

[1] Ekert A, Jozsa R (1996). Quantum Computation and Shor’s Factoring Algorithm. Rev Mod Phys.

[2] Nielsen MA, Chuang I (2000). Quantum Information and Quantum Computation.

[3] Deutsch D (1985). Quantum Theory, the Church-Turing Principle and the Universal Quantum Computer. Proc Royal Soc Lond A.

[4] Deutsch D, Jozsa R (1992). Rapid Solution of Problems by Quantum Computation. Proc Royal Soc Lond A.

[5] Chi DP, Kim J, Lee S (2001). Initialization Free Generalized Deutsch-Jozsa. J Phys A - Math Gen.

[6] Gudder S (2003). Quantum Computation. Amer Math Monthly.

